# Clinical Comparison of Articulating Paper, Intraoral Scanner, and Jaw-Tracking Device for Occlusal Analysis

**DOI:** 10.3390/jcm15197587

**Published:** 2026-09-30

**Authors:** Manuela-Maria Manziuc, Mara Mihaela Savu, Flavia Groza, Ștefan Cristian Vesa, Cosmin Ifrim, George Burian, Maria Jarda, Manuela Tăut, Ana Ispas, Daniela Popa, Smaranda Buduru

**Affiliations:** 1 Prosthetic Dentistry and Dental Materials Department, Iuliu Hațieganu University of Medicine and Pharmacy, 32 Clinicilor Street, 400006 Cluj-Napoca, Romania; manuelamanziuc@yahoo.com (M.-M.M.);; 2 Department of Pharmacology, Toxicology and Clinical Pharmacology, Iuliu Haţieganu University of Medicine and Pharmacy, 400337 Cluj-Napoca, Romania

**Keywords:** digital dentistry, maximum intercuspation, articulating paper, intraoral scanner, jaw-tracking system

## Abstract

Recent advances in digital dentistry have transformed occlusal analysis through the introduction of new digital workflows for recording static and dynamic occlusal contacts. This clinical study aimed to evaluate the reliability and concordance of two digital clinical workflows, the Medit i700 intraoral scanner and the Tizian JMA-Optic jaw-tracking system, using 100 μm articulating paper as the reference comparator for static and dynamic occlusal-contact recordings. **Materials and Methods**: Twenty-six participants aged 20 to 25 years were included in this study. For each participant, static and dynamic occlusal contacts were recorded in maximum intercuspation position (MIP), protrusive, and right and left laterotrusive movements, using 100 μm articulating paper, Medit i700, and Tizian JMA-Optic system. Intra-rater reliability was assessed using ICC(A,1) for the quantitative number of MIP contacts and Cohen’s kappa for dynamic occlusal-contact recordings. Sensitivity, specificity, positive predictive value, and negative predictive value of the digital systems were evaluated relative to the articulating-paper reference comparator. Agreement between articulating paper and the two digital workflows was assessed using the intraclass correlation coefficient for the quantitative variables. **Results**: Intra-rater reliability was generally high but varied according to the recording method and occlusal-contact category. Overall, the Medit i700 showed numerically higher sensitivity and positive predictive value point estimates in several dynamic occlusal-contact categories than Tizian JMA-Optic, while specificity and negative predictive values were generally high for both systems. Both digital workflows showed good overall agreement with articulating paper in recording the number of occlusal contacts in MIP. Agreement was good at arch level, poor in the anterior regions, and moderate in the posterior regions. **Conclusions:** Both digital workflows showed generally high intra-rater reliability for dynamic occlusal recordings, with reliability varying according to the occlusal-contact category. Both systems demonstrated good agreement with the articulating-paper reference comparator for quantifying occlusal contacts in MIP, although substantial differences in measured contact area were observed. The numerical differences between Medit i700 and Tizian JMA-Optic should be interpreted descriptively rather than as evidence of superiority of either workflow. Although the digital systems provide valuable quantitative information for occlusal analysis, articulating paper remains a widely used conventional method for clinical identification of occlusal contacts and served as the reference comparator in the present study.

## 1. Introduction

In recent years, innovations in digital technology have significantly influenced the development of various dental specialties, particularly prosthodontics [1]. The modern treatment concepts, defined by minimally invasive approaches, involve a large variety of advanced restorative solutions aimed to preserve the integrity of dental hard tissues [2]. However, the longevity of the dental restorations mainly depends on achieving harmonious occlusal relationships [3].

Although manufacturers continue to develop innovative digital devices capable of recording and evaluating the occlusal contacts, there is still no consensus in the literature regarding an ideal, objective, or reference method for the accurate assessment of static and dynamic occlusal status [4,5].

Articulating paper represents one of the most widely used methods for marking both the static occlusal contact points in maximum intercuspation position (MIP) and the guidance pathways during protrusive and laterotrusive mandibular movements [6,7]. The accuracy of occlusal assessment using articulating paper is influenced by several factors, such as its physical properties, defined by the material composition, elasticity, and thickness [8]. Overall, material thickness represents an important determinant of occlusal assessment, as thicker materials may generate a greater number and size of occlusal contacts markings, and may alter their distribution compared with thinner materials [9]. When the thickness of the interposed occlusal registration material exceeds the available interocclusal space, false-positive occlusal contacts may be recorded, and independently may alter the the mandibular closure movement. However, previous comparative studies have used articulating paper of varying thicknesses as reference for occlusal assessment, including 8 μm foil [10], 40 μm [11], 80 μm [12], and 100 μm articulating paper [13,14]. Nevertheless, the clinical evaluation of the occlusal contacts relies on clinician interpretation, which may introduce a degree of subjectivity [8,15,16]. Another limitation of the articulating paper is represented by its inability to sequentially record the presence of the occlusal contacts between antagonistic teeth, and to provide information related to the contact pressure level [17]. Despite its recognized limitations, articulating paper remains the reference method for evaluating the occlusal contacts, as it represents the most widely used conventional technique in the daily practice, being frequently referred as the reference method in comparative studies [18]. Yet, comparisons between physical and digital occlusal-recording methods should be interpreted in relation to the specific properties of the selected physical indicator [8].

To improve the objectivity of occlusal assessment, manufacturers have developed various digital systems with integrated software designed for the recording and evaluation of occlusal contacts [1].

The introduction of intraoral scanners (IOSs) has significantly transformed the clinical workflow in dentistry. IOS systems allow the digital acquisition of the maxillary and mandibular dental arches and the generation of virtual occlusion by recording the maxillomandibular relationship and precisely aligning the digital arch scans. With the introduction of new software versions, certain IOS systems have the capacity to record and analyze the occlusal contacts during the dynamic mandibular movements, including protrusion and laterotrusion [19]. Among these systems, the Medit i700 IOS (MEDIT, Seoul, Republic of Korea) includes two dedicated software functions, the Medit Occlusion Analyzer and Mandibular Movement Mode (Medit Scan for Clinics, MEDIT, Seoul, Republic of Korea), which facilitate the virtual evaluation of static and dynamic occlusal contacts, including their number, distribution, and interocclusal proximity displayed as a color-coded virtual occlusogram [20]. These color-coded representations indicate geometric interocclusal proximity and should not be interpreted as direct measurements of occlusal force or pressure intensity [21].

Previous studies have suggested that the continuous development of the IOS technology may improve the accuracy of the occlusal evaluation [22,23,24]. Fraile et al. [10] reported that Trios Color POD intraoral scanner (Phibo, 3 Shape, Copenhagen, Denmark) showed greater validity than the T-Scan III system (Tekscan, Boston, MA, USA), when compared with 8 µm articulating paper. Similar results were obtained by Cao et al. [25], who reported higher accuracy for Cerec Omnicam system (Cerec Omnicam, Sirona, Germany) than for the T-Scan system, using the articulating as the reference method. Comparisons of T-Scan and IOS-based occlusal analysis have also demonstrated that the systems provide different occlusal measurements. In an in vivo comparison with CEREC Omnicam, T-Scan showed greater sensitivity under the tested conditions, while the IOS system did not provide a direct measurement of occlusal force. Therefore, differences in the extent or color-coded representation of contacts between these technologies should not be interpreted as simple measurement error without considering the parameter represented by each system [26].

However, the computerized occlusal-analysis systems such as T-Scan differ from both articulating paper and IOS-based occlusograms. T-Scan system is designed to record relative occlusal force distribution and timing during tooth contact, whereas articulating paper produces physical ink markings and IOS occlusograms primarily provide geometric information on virtual contact or interocclusal proximity. Therefore, lack of correspondence in displayed contact area among these systems should not, by itself, be interpreted as evidence of inaccuracy [10,21].

To address the limitations associated with the recording of the occlusal contacts during mandibular movements, digital jaw-tracking devices have been introduced. These innovative devices use photometry, infrared, or ultrasonic technologies to capture the mandibular motion, including the maxillomandibular relationships, and excursive movements, and to transfer the data into CAD software programs, having the capacity to improve the clinical assessment and increase the precision of restorative and prosthetic treatments [1]. However, the accuracy of the jaw-tracking systems has not yet been fully studied and clarified [27,28]. The Tizian JMA-Optic system (Zebris Medical GmbH, Isny, Germany, distributed by Schütz Dental GmbH) is an infrared optical tracking devices that uses a facebow with integrated cameras to capture and analyze the movements of the sensor positioned on the mandibular arch [1]. Therefore, the clinician can evaluate the dental contacts in both static and dynamic occlusion.

Although several studies have evaluated different IOSs devices and jaw-tracking systems for recording centric relation [29,30,31,32], there is still limited evidence regarding their ability to accurately record and assess the occlusal status. Wong King et al. [11] performed an in vitro comparison of articulating paper with various types of occlusal analysis devices, including Occlusense (Bausch), Trios 3 and Tios 5 (3Shape), Primescan (Dentsply Sirona) and Modjaw (Modjaw), during MIP, protrusive and right laterotrusive mandibular movements. They concluded that none of the studied digital system completely replicated the articulating paper. In MIP, Trios and Primescan tended to overestimate the contacts, IOSs performed poorly during dynamic occlusal assessment, and Modjaw-integrated systems showed the highest overall concordance. A different perspective on digital occlusal assessment has been provided by Karaca et al. [33], who investigated the influence of head position on dental contacts registration in MIP using the TRIOS4 intraoral scanner (TRIOS4, 3Shape A/S) and the JMA-Optic system (Zebris Medical GmbH, Isny, Germany). Their results indicated no significant differences in the overall location, intensity, and number of occlusal contacts among neutral, forward and backward head positions. However, compared with the neutral head position, the occlusal contact location differed in forward and backward position, and the contact number increased with greater head inclination.

Conventional and digital occlusal-analysis methods are based on different physical and methodological principles and therefore should not be interpreted as measuring identical occlusal parameters. Articulating paper identifies contact areas through ink transfer, and the resulting markings may be influenced by the strength, elasticity, and thickness of the material, as well as by substrate characteristics or saliva moisture [8,17]. Moreover, the size of the articulating paper markings, representing the apparent contact area, is influenced by the material thickness [34]. Intraoral scanner-based occlusograms provide a virtual representation of occlusal contacts or interocclusal proximity derived from the geometric relationship between digitally acquired dental arches. Jaw-tracking systems combined with virtual dental models additionally reproduce mandibular movement trajectories over time. Neither IOS-based occlusograms nor optical jaw-tracking systems directly measure interocclusal force or pressure [21]. Therefore, comparisons among these methods should be interpreted primarily in terms of contact representation or concordance, rather than as evidence of equivalence between the physical quantities measured.

Despite the growing clinical availability of the IOSs devices and jaw-tracking systems, comparative information regarding their performance relative to articulating paper in the assessment of static and dynamic occlusal contacts is still lacking [27,32,35].

This clinical study aimed to evaluate the reliability and concordance of two digital clinical workflows, the Medit i700 intraoral scanner and the Tizian JMA-Optic jaw-tracking system, using 100 μm articulating paper as the reference comparator for static and dynamic occlusal-contact recordings.

The objectives of this in vivo clinical study were: (1) to evaluate the intra-rater reliability of articulating paper, Medit i700, and Tizian JMA-Optic for static and dynamic occlusal recordings; (2) to assess the tooth-level concordance between the Medit i700 and Tizian JMA-Optic with 100 μm articulating paper for the identification of dynamic occlusal-contact; and (3) to evaluate the agreement between the digital workflows and articulating paper in the quantitative assessment of the number of occlusal contacts recorded in maximum intercuspation position.

## 2. Materials and Methods

Twenty-six participants, including 9 males and 17 females, were recruited between December 2025 and April 2026. All participants were students of the Faculty of Dental Medicine, “Iuliu Hațieganu” University of Medicine and Pharmacy, Cluj-Napoca, Romania. For this study, no formal sample-size calculation was performed; therefore, the sample size was determined by the feasibility of recruiting participants who met the following inclusion criteria: age between 20 and 25 years, complete permanent dentition, stable and functional occlusal status, and healthy temporomandibular joints. Exclusion criteria included severe crowding, dental wear and partial edentulism. Before performing any clinical procedure related to the study, all participants provided written informed consent, approved by the University’s Ethical Committee (No. 382/19.12.2025).

Three methods for recording static and dynamic occlusal contacts were evaluated in maximum intercuspation position (MIP), protrusion, and right and left laterotrusion, on both the working and non-working sides, by using the 100 μm articulating paper-considered the reference method, an intraoral scanner, and a jaw-tracking system. For each participant, all occlusal recordings were performed by one experienced clinician (M.M.M.) on the same day, using the three occlusal recording methods in the following sequence: articulating paper, intraoral scanner, and jaw-tracking system. Each occlusal recording was scheduled one hour apart. To assess the reproducibility of the recordings and to avoid the muscular fatigue, two independent recording sessions were performed for each participant by the same investigator, scheduled one week apart.

### 2.1. Articulating Paper

The 100 μm articulating paper (Dr Jean Bausch GmbH & Co KG, Koln, Germany) was selected for marking occlusal contacts, due to its widespread clinical use and its ability to provide clearly visible markings that can be assessed during both static and dynamic occlusal evaluation. The material was selected to reproduce a conventional clinical workflow rather than to represent a diagnostic gold standard.

**Figure 1:**
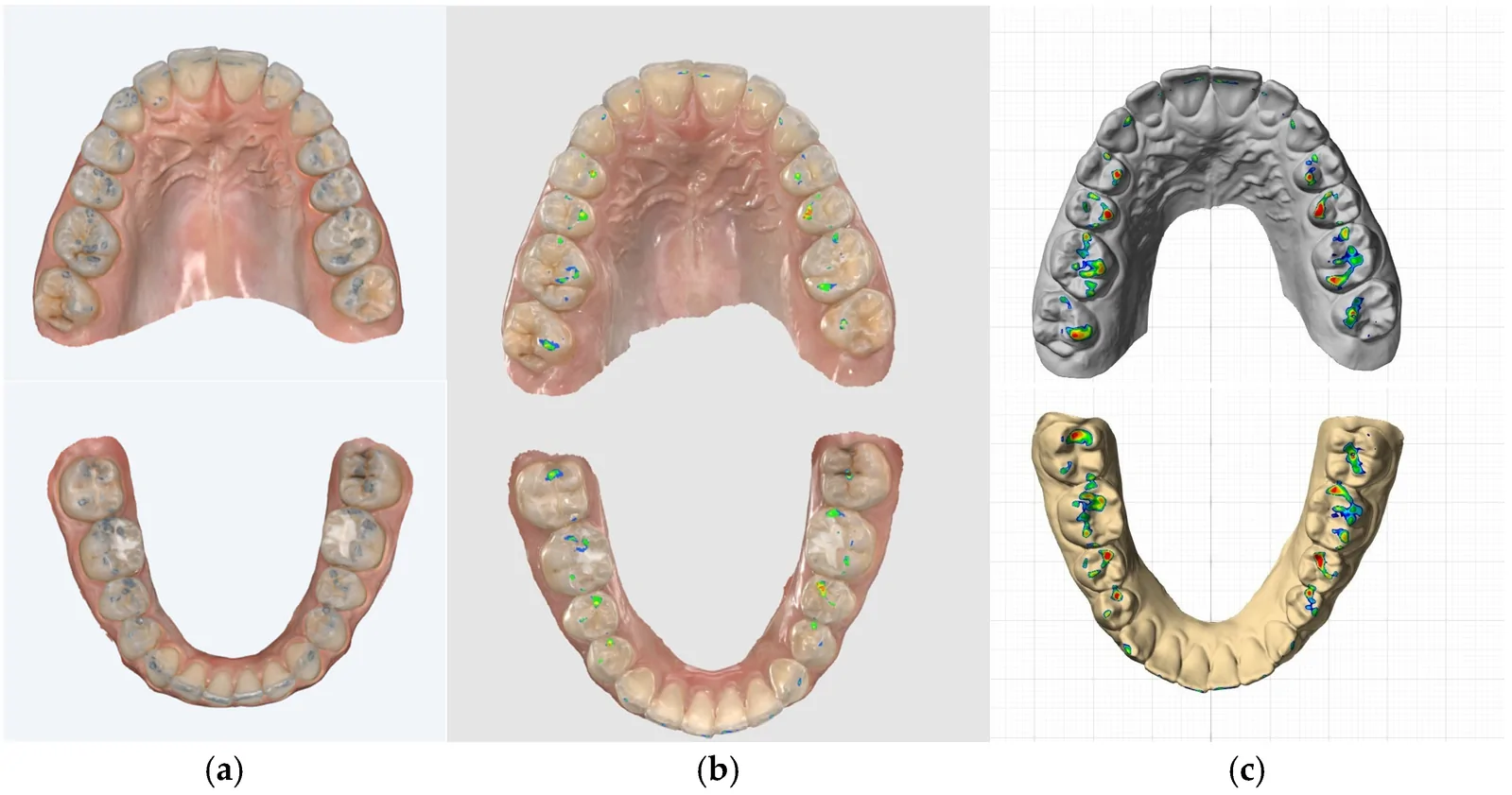
Representative occlusal-contact recordings obtained in MIP using (**a**) 100 μm articulating paper, (**b**) Medit i700, and (**c**) Tizian JMA-Optic. The three workflows generate different visual representations of occlusal contact. Correspondence at tooth level does not necessarily indicate exact spatial overlap of individual contact locations.

Each participant was comfortably seated in the dental chair at a 45-degree angle [36,37], with the head supported by the headrest to maintain the Frankfort plane parallel to the floor. This standardized head position was maintained while performing all the occlusal recordings, regardless of the technique. Prior to data collection, to ensure the reproducibility of mandibular position and movement, each participant was trained to perform opening and closing movements in MIP, protrusive, right and left laterotrusive movements. To mark the static dental contacts, all the teeth were cleaned and dried, and participants were asked to bite in MIP on blue articulating paper positioned bilaterally between the two antagonistic dental arches (Figure 1a).

Protrusive (P) guidance was defined as functional when the central incisors (CInc), either alone or in combination with the lateral incisors (LI), provided symmetrical guidance along their marginal ridges. The laterotrusive (L) occlusal schemes were classified according to the teeth that guided the mandibular excursion in transversal plane. Canine guidance (CG) was defined as the guidance performed exclusively by the canines; group function (GF) represented the guidance involving the canine, premolars and molars; and anterolateral guidance (AG) as the guidance performed by the canine in combination with the lateral incisors (LI), either alone or with the central incisors (CInc). To evaluate the presence of nonfunctional contacts associated with both protrusive and laterotrusive guidances, the passive occlusal interference was defined as any dental contact between antagonistic teeth occurring on the non-working side during the mandibular excursions. Therefore, the passive propulsive interference (PPI) was represented by nonfunctional dental contacts occurring on the posterior teeth during protrusive movement, whereas the passive laterotrusive interference (PLI) was defined as any contact between antagonistic teeth on the non-working side during right or left laterotrusive movement.

**Figure 2:**
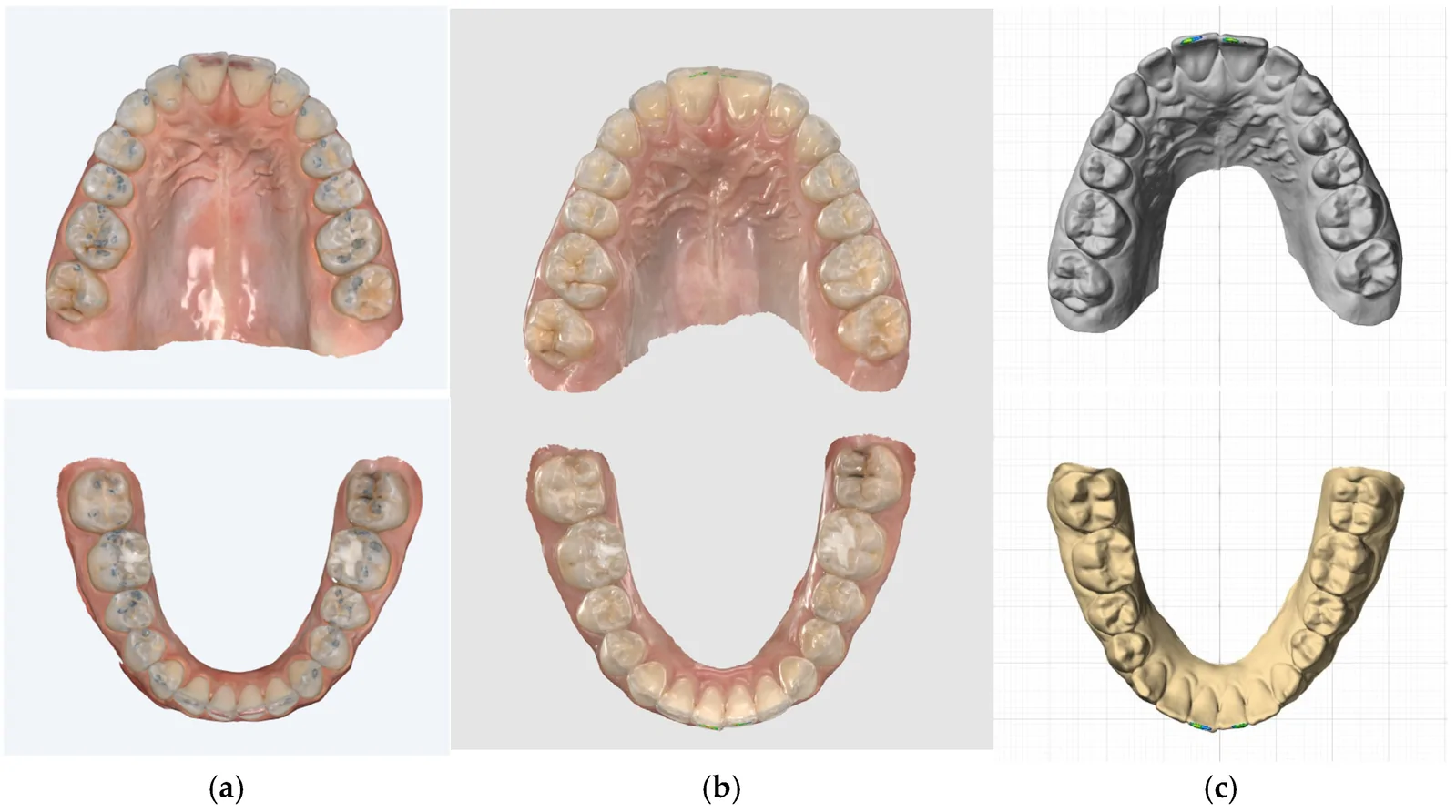
Assessment of protrusive guidance (P) performed by the central incisors (CI) using (**a**) 100 μm articulating paper, (**b**) Medit i700 and (**c**) Tizian JMA-Optic system.

After the registration of the occlusal contacts and removal of the articulating paper, digital impressions of the upper and lower dental arches were acquired using an intraoral scanner (Medit i700, MEDIT, Seoul, Republic of Korea) (Figure 2a).

### 2.2. Intraoral Scanner

The maxillary and the mandibular dental arches were scanned using an intraoral scanner (Medit i700, MEDIT, Seoul, Republic of Korea) in accordance with the manufacturer’s instructions. To ensure consistency in the scanning process, the investigator followed a strict protocol involving scanner calibration, prior to data acquisition for each participant. During scanning, the dental chair was reclined with a 45-degree angle [36,37], and participants were asked to close in MIP. Each virtual interocclusal record was performed bilaterally, on both right and left hemiarches, with half of the scanning area covering the buccal surfaces of the maxillary teeth, and the other half included the mandibular teeth, extending from the first molars to the canines [38]. To maintain the accuracy of the digital recordings, cutting off, rescanning or overlapping procedures were avoided.

Using a dedicated software program (Medit Scan for Clinics v3.4.12, MEDIT, Seoul, Republic of Korea), the maxillary and the mandibular digital models were aligned in MIP. Afterward, the participants were asked to perform the protrusive and left and right laterotrusive movements, which were digital recorded. The presence or absence of the occlusal contacts in MIP was assessed using the The Medit Occlusion Analyzer software function (Medit Scan for Clinics v3.4.12, MEDIT, Seoul, Republic of Korea). In addition, the functional and non-functional occlusal contacts in protrusion and laterotrusion were evaluated using the Mandibular Movement Mode (Medit Scan for Clinics v3.4.12, MEDIT, Seoul, Republic of Korea) (Figure 1b and Figure 2b).

### 2.3. Jaw-Tracking System

Static and dynamic occlusal recordings were also obtained using a jaw-tracking system, the Tizian JMA-Optic system (Zebris Medical GmbH, Isny, Germany, distributed by Schütz Dental GmbH). The digital facebow was positioned on the patient head according to the manufacturer’s recommendations, parallel to the interpupillary line from frontal view, and parallel to the Frankfurt plane from lateral view. The mandibular fork was attached on the buccal surfaces of the mandibular teeth using bis-acrylic autopolymerizing composite resin (Protemp, 3M ESPE, St. Paul, MN, USA). To perform the recordings in sequence with the software protocol, the facial cutaneous and temporomandibular joint reference points were selected and recorded. An alignment fork was used to record the three-dimensional position of the maxilla to the base of the cranium. Optical scans of the maxillary and mandibular dental arches acquired using Medit i700 IOS were exported as “.ply” files and imported into Tizian Function Pro 4.0.2 software (Schütz Dental GmbH, Rosbach vor der Höhe, Germany). The digital models were automatically aligned in MIP based on the virtual interocclusal recording provided by the Medit i700 software. The position of the mandible to the maxilla was recorded in MIP, by asking each participant to perform three repeated opening and closing movements ending in MIP. Afterward, the participants were asked to perform the protrusive and left and right laterotrusive movements, which were recorded in real time. Like the protocol used for the IOS, functional and non-functional occlusal contacts were evaluated in protrusion and laterotrusion using the Tizian Function Pro 4.0.2 software (Schütz Dental GmbH, Rosbach vor der Höhe, Germany) (Figure 1c and Figure 2c).

For each participant, all conventional and digital occlusal recording methods were performed by the same investigator (M.M.M.), in two independent sessions scheduled one week apart. In this study, the occlusal contacts were analyzed as dichotomy variable (yes/no) defined by the presence or absence of a dental contact for each tooth, regardless of the location, size or intensity of the contact [10,18,39]. The study was designed to assess tooth-level contact detection and concordance between recording methods, rather than spatial agreement in the exact intra-tooth location of individual occlusal contacts. To accurately identify and quantify occlusal contacts in MIP, all relevant anatomic landmarks of all permanent teeth were evaluated, excluding the third molars, due to their considerably variability in presence, eruption status and morphology, which could introduce additional variability among study participants.

For between-method comparisons, only data from the 1st recording session were used. The 2nd recording session was used only for the assessment of intra-rater reliability. Occlusal-contact presence or absence was evaluated at tooth level; however, because multiple teeth were assessed within each participant, tooth-level observations were considered clustered within participants for inferential analyses.

The statistical analyses were conducted using R software (R Foundation for Statistical Computing, Vienna, Austria), version 4.6.0. Data were imported and managed using the readxl and dplyr packages. Dichotomous occlusal-contact variables were defined according to the presence or absence of contact between teeth. Intra-rater reliability for dichotomous dynamic occlusal-contact recordings and agreement between the digital recording methods and the 100 μm articulating paper were computed using Cohen’s kappa statistic. Dynamic intra-rater reliability was assessed by comparing tooth-level observations from the two recording sessions. Because multiple teeth were evaluated within each participant, 95% confidence intervals for Cohen’s kappa were obtained using participant-level cluster bootstrap resampling with 10,000 replicates, with all tooth-level observations from each sampled participant retained together. Confidence intervals were reported as not estimable (NE) when a cluster-adjusted interval could not be reliably estimated because of complete concordance or insufficient between-participant information in a sparse category. Only data from the first recording session were analyzed for between-method comparisons; data from the second recording session were used exclusively for the assessment of intra-rater reliability. The presence or absence of an occlusal contact was recorded for each tooth. Because multiple tooth-level observations were obtained within each participant, observations were considered clustered within participants for inferential analyses. The 100 μm articulating paper served as the reference comparator for calculating sensitivity, specificity, positive predictive value (PPV), and negative predictive value (NPV) for Medit i700 and Tizian JMA-Optic. Point estimates were calculated at tooth level, while 95% confidence intervals were estimated using a cluster-adjusted design with the participant as the clustering unit, using the beta method implemented in the R survey package. Confidence intervals were reported as not estimable (NE) when the corresponding proportion showed complete concordance or when there was insufficient between-participant information to estimate a cluster-adjusted confidence interval in a sparse category. The underlying numbers of true positives (TP), false negatives (FN), false positives (FP), and true negatives (TN) were also reported. Cohen’s kappa was retained as a descriptive measure of tooth-level agreement between methods.

For MIP quantification intra-rater reliability of tooth-level MIP contact counts, ICC(A,1) estimates were generated for within-rater MIP contact counts from first and second recordings of tooth observations. Multiple teeth were measured per subject, so 95% confidence interval estimates were calculated from subject-cluster bootstrap resampling (B = 10,000), taking within-subject observation sets as an intact group. ICC(A,1) was used to assess agreement between the articulating-paper reference comparator and each digital workflow for the quantitative number of MIP contacts recorded at tooth level, using a two-way mixed-effects model for absolute agreement based on single measurements. Because multiple tooth-level observations were obtained from each participant, 95% confidence intervals were estimated using participant-level cluster bootstrap resampling with 10,000 replicates, retaining all tooth-level observations from each sampled participant together. The overall analysis included all teeth, while the maxillary and mandibular arches and the anterior and posterior regions within each arch were analyzed separately. ICC values were interpreted as poor (<0.50), moderate (0.50 to <0.75), good (0.75 to 0.90), and excellent (>0.90).

## 3. Results

Intra-rater reliability for the number of occlusal contacts recorded in MIP was assessed using ICC(A,1). Excellent intra-rater reliability was observed for articulating paper (ICC = 0.939, 95% CI: 0.917–0.956) and Tizian JMA-Optic (ICC = 0.936, 95% CI: 0.911–0.953), while Medit i700 showed good intra-rater reliability (ICC = 0.894, 95% CI: 0.874–0.911). The 95% confidence intervals were obtained using participant-level cluster bootstrap resampling, with all tooth-level observations from each sampled participant retained together.

Intra-rater reliability for dynamic occlusal recordings was assessed using Cohen’s kappa. Kappa point estimates were generally high but varied according to the occlusal-contact category and recording method. Several categories showed complete concordance, whereas wide participant-level cluster-bootstrap confidence intervals were observed for some uncommon dynamic occlusal patterns, reflecting limited precision in these sparse categories (Table 1).

Sensitivity represented the proportion of occlusal contacts marked by the 100 μm articulating-paper reference method that were also identified by each digital workflow. Sensitivity point estimates varied by type of dynamic occlusal contact, and more categories showed numerically higher values for Medit i700 than for Tizian JMA-Optic, although several guidance and interference patterns were rare at the participant level despite the larger number of tooth-level observations. This resulted in wide participant-cluster-adjusted confidence intervals for many point estimates; several sparse categories were not estimable (Table 2). Specificity represented the proportion of teeth without an occlusal contact according to the articulating-paper reference method that were also classified as contact-negative by each digital workflow. For most categories, specificity point estimates remained high in both digital workflows, although the precision of individual estimates varied after accounting for clustering of teeth within participants (Table 2).

The PPV refers to the probability that a contact identified by the digital workflow corresponds to a contact marked by the 100 μm articulating-paper reference method. The point estimates for PPV differed substantially between dynamic occlusal-contact categories and estimates for Medit i700 were generally numerically higher than those for Tizian JMA-Optic, although the opposite was observed for some categories. Some point estimates had wide cluster-adjusted confidence intervals, reflecting substantial imprecision because positive contacts in these categories were represented in only a small number of participants (Table 3).

The NPV refers to the probability that a tooth classified as contact-negative by the digital workflow was also contact-negative according to the 100 μm articulating-paper reference method. NPV point estimates were high across all dynamic occlusal-contact categories for both digital workflows, ranging from 91.82% to 100%. After accounting for within-participant clustering, the confidence intervals varied in width according to the amount of independent participant-level information available.

All teeth: Both digital workflows showed good agreement with the articulating-paper reference comparator for the quantitative number of occlusal contacts recorded in MIP. The ICC was 0.784 (95% CI: 0.749–0.812) for Medit i700 and 0.756 (95% CI: 0.709–0.792) for Tizian JMA-Optic.

Maxilla/Mandible: Good agreement with the articulating-paper reference comparator was observed for both digital workflows at arch level. For the maxillary arch, the ICC was 0.789 (95% CI: 0.743–0.826) for Medit i700 and 0.752 (95% CI: 0.697–0.794) for Tizian JMA-Optic. For the mandibular arch, the corresponding ICCs were 0.779 (95% CI: 0.739–0.813) and 0.760 (95% CI: 0.708–0.804), respectively.

Anterior/Posterior Areas: Area-specific analyses demonstrated poor agreement in the anterior areas and moderate agreement in the posterior areas. For Medit i700, agreement with the articulating-paper reference comparator was poor in both the maxillary anterior area (ICC = 0.460, 95% CI: 0.297–0.597) and the mandibular anterior area (ICC = 0.196, 95% CI: 0.048–0.346). Similarly, for Tizian JMA-Optic, agreement was poor in the maxillary anterior area (ICC = 0.432, 95% CI: 0.251–0.575) and mandibular anterior area (ICC = 0.201, 95% CI: 0.062–0.351). In the posterior areas, agreement was moderate for Medit i700 in both the maxillary (ICC = 0.685, 95% CI: 0.610–0.744) and mandibular areas (ICC = 0.664, 95% CI: 0.597–0.721), and for Tizian JMA-Optic in the maxillary (ICC = 0.627, 95% CI: 0.534–0.695) and mandibular areas (ICC = 0.636, 95% CI: 0.545–0.709).

## 4. Discussion

The development of digital technologies has reshaped prosthetic and restorative dentistry by improving clinical workflow and enhancing dental occlusal assessment. Although innovative devices and new software are increasingly used to record the static and dynamic occlusal status, articulating paper remains the most widely used clinical method to mark and evaluate the dental contacts [18]. Despite its limitations, articulating paper is also considered the reference method to evaluate the accuracy and validity of digital occlusal analysis systems [1,40].

This in vivo study aimed to compare two different digital occlusal analysis workflows with articulating paper in the assessment of both static occlusal contacts in MIP, and dynamic occlusal contacts during protrusive and right and left laterotrusive movements.

According to our results, all three recording methods demonstrated high intra-rater reliability for both static and dynamic occlusal-contact recordings, although the degree of reliability varied among the methods. For static occlusal-contact recordings in maximum intercuspation position (MIP), articulating paper and Tizian JMA-Optic demonstrated excellent reliability, whereas the Medit i700 showed good reliability. For dynamic recordings, kappa point estimates were generally high but varied according to the occlusal-contact category and recording method, without a consistent ordering of the three methods across categories. These findings suggest that both digital workflows can provide reproducible occlusal-contact recordings, although reproducibility may vary according to the recording method and the type of occlusal assessment.

The high reliability observed with articulating paper may be associated with its standardized clinical application, which minimizes the measurement variability and allows direct visualization of the occlusal contact marks, facilitating their consistent identification and evaluation by clinicians. In contrast, digital occlusal assessment involves multiple sequential clinical procedures, including standardized data acquisition, accurate recording of the static mandibular position and dynamic mandibular movements, and software-based processing to generate the virtual representation of the occlusal status. Therefore, sources of variability associated with any stage of the digital workflow may influence the reproducibility of repeated measurements.

In the present study, to minimize the potential sources of variability and ensure consistency during scanning, the clinician followed a standardized protocol that included Medit i700 IOS calibration, controlled ambient conditions, and avoidance of cutting off, rescanning or overlapping procedures [23]. Nevertheless, the minor differences observed between the repeated digital occlusal recordings may have been determined by the software algorithms used to align of the maxillary and mandibular digital dental arches in MIP and to record and process the mandibular movement. In addition, operator-dependent factors, including the scanner distance and angulation, as well as saliva control, may have influenced the reproducibility of the recordings [23,24].

The Tizian JMA-Optic system uses stereoscopic technology to track the mandibular movements in real time. Two cameras mounted on the facebow detect the infrared light-emitting diode (LED) pattern generated by a sensor positioned on the mandibular dental arch. Changes in the spatial position of the sensor during opening, closing, protrusive and laterotrusive movements are recorded and processed by the integrated software to generate three-dimensional trajectory of the mandibular movements [28]. The higher intra-rater reliability observed with the Tizian JMA-Optic workflow compared with the Medit i700 may be related to the standardized acquisition and processing of dynamic mandibular movements data. Unlike articulating paper, which provides a direct intraoral assessment of the occlusal status, the digital systems offer an indirect evaluation of dental occlusion. When using Tizian JMA-Optic, minor variations in facebow and mandibular sensor positioning, as well as in data processing, may influence the reproducibility of the recordings [28]. However, the Tizian JMA-Optic workflow was not completely independent of the Medit i700 workflow, as Tizian Function Pro 4.0.2 software aligned the digital models in MIP using the virtual interocclusal record obtained with the Medit Scan for Clinics v3.4.12 software.

Furthermore, patient-related factors may also contribute to variability, particularly the ability to reproduce mandibular functional movements with consistent trajectory, velocity, and amplitude. Despite these potential sources of variability, our results demonstrated high intra-rater reliability for the dynamic occlusal-contact recordings, suggesting that the Tizian JMA-Optic can provide reproducible occlusal recordings when a standardized clinical protocol is followed by the same clinician.

For static occlusal evaluation in MIP, articulating paper and the Tizian JMA-Optic demonstrated excellent intra-rater reliability, while Medit i700 showed good reliability. This difference may be attributed, at least in part, to physiological variations during repeated mandibular closure in MIP. In addition, the digital data acquisition and integration procedures of the Tizian JMA-Optic workflow may contribute to the reproducibility of dental contact recordings [28].

Similar findings were reported by Manziuc et al. [39], who evaluated the inter-rater reliability of occlusal contacts assessment using 40 µm articulating paper, the Medit i700, and OccluSense. They reported higher inter-rater reliability for articulating paper than for the two digital devices, when evaluating both the static and dynamic occlusal contacts. Similarly, Wong King el al [11] compared the performance of different digital occlusal analysis systems, including Occlusense (Bausch), Trios 3, Trios 5, and Primescan, used either independently or in combination with Modjaw (Modjaw), with 40 µm articulating paper (Bausch Arti-check) for evaluating dental contacts in MIP, protrusion and right laterotrusion. Consistent with our findings, they reported higher intra- and inter-examiner reliability for static occlusal recordings obtained with articulating paper than with intraoral scanning systems. However, for dynamic occlusal evaluation, their results indicated higher ICC values for digital devices compared with articulating paper.

The tooth-level concordance analysis revealed variability in the sensitivity and specificity for both digital devices for the assessment of dynamic occlusal contacts. Overall, the Medit i700 showed numerically higher sensitivity point estimates than the Tizian JMA-Optic system, when compared with 100 μm articulating paper. The variation in sensitivity across the evaluated dynamic occlusal-contact categories may be related to differences in the ability of the two digital workflows to identify specific occlusal patterns.

The high sensitivity point estimates observed with the Medit i700 may be related to its digital workflow, whereas the Tizian JMA-Optic involves more complex clinical and digital procedures that may introduce additional sources of variability. Previous studies have shown that the Medit i700 IOS has the capacity to accurately record the maxillomandibular relationship and to align the maxillary and mandibular virtual models in MIP [1,22,41], which represents the reference mandibular position from which protrusive and laterotrusive movements are performed. Inaccuracies in the virtual alignment of the dental models in MIP, as well as variations in recording the trajectory of the dynamic mandibular movements, may affect the ability of the digital device to accurately record the occlusal contacts during protrusion and laterotrusion. Therefore, several factors may influence the accuracy of occlusal assessment with intraoral scanners, including the technique used to record the MIP, the number, size and location of the dental contacts, the length of the dental arches, the hardware and software performance, and integrated software functions such as such as Mandibular Movement Mode (Medit Scan for Clinics, Medit, Seoul, Republic of Korea) [23,24].

The high positive predictive point estimates observed for the Medit i700 suggest greater concordance with articulating paper in identifying dynamic occlusal contacts than those observed for the Tizian JMA-Optic system. However, the two digital workflows are not completely independent, as Tizian JMA-Optic workflow uses the digital models and virtual interocclusal record acquired with the Medit i700 IOS.

Nevertheless, both digital workflows presented high specificity point estimates. These findings suggest that both systems had a high capacity to identify the absence of occlusal contacts relative to the articulating paper, rather than confirming their presence. Moreover, the consistently high negative predictive value point estimates across all dynamic occlusal-contact categories for both digital workflows indicate a high probability of identifying the absence of the occlusal contacts.

A previous study had concluded that integrating the Modjaw jaw-tracking system with Trios 3 (3Shape) intraoral scans improved the accuracy of the dynamic occlusal analysis, during both protrusive and right laterotrusive movements [11]. However, the evidence regarding the use of jaw-tracking systems for dynamic occlusal assessment remains limited. Further research is needed to compare the accuracy, reliability and clinical performance of different jaw-tracking systems, as well as their integration with various intraoral scanners systems.

Our findings indicated that both digital workflows showed good agreement with the articulating-paper reference comparator for the number of occlusal contacts recorded in MIP, with the Medit i700 showing a higher ICC point estimate than Tizian JMA-Optic. ICC point estimates were generally numerically higher for Medit i700, except in the mandibular anterior area, where the Tizian JMA-Optic estimate was slightly higher. These numerical differences were not formally tested as between-workflow differences. These findings suggest that agreement in the number of occlusal contacts may vary according to the dental region and the characteristics of the digital workflow.

The lower agreement observed in the anterior area may be related to the smaller number of occlusal contacts identified in this region. Posterior teeth have more complex occlusal morphology, including cusps, inclines, marginal ridges, and fossae, which may result in a greater number and larger area of occlusal contacts and influence the digital occlusal contact recording. Thus, tooth morphology and the distribution and number of occlusal contacts may influence the differences in agreement between the two digital workflows across anterior and posterior areas.

Nevertheless, agreement at the tooth level does not necessarily indicate spatial agreement of the corresponding occclusal contacts. As illustrated by the representative images, contacts identified on the same tooth by different methods may differ in their spatial distribution. This difference may arise from the distinct physical and computational principles of each occlusal recording method, including ink transfer and indicator properties for articulating paper, geometric approximation of opposing surfaces for the IOS workflow, and virtual reconstruction of mandibular movements for the jaw-tracking workflow. However, the characteristics of the articulating paper used as the physical reference comparator should be considered when interpreting these findings. The 100 μm articulating paper may produce broader or additional markings than thinner indicators, particularly when the interposed material modifies the effective relationship between opposing occlusal surfaces. Consequently, the higher concordance observed in the posterior area may partly reflect the thickness and physical behavior of the articulating paper, rather than indicating superior performance of either digital workflow. Moreover, the present tooth-level, count-based analysis was not intended to support clinical occlusal diagnosis or selective occlusal adjustment. Similar numbers the contacts or concordance in the presence of a contact on the same tooth do not necessarily indicate that the methods identified the same intra-tooth contact locations. This approach does not provide information regarding the magnitude or timing of contact forces. Therefore, these findings should be interpreted as a methodological assessment of toot-level contact detection and reproducibility rather than as direct guidance for clinical occlusal treatment.

Furthermore, maximum intercuspation position (MIP) may not be completely reproducible within the same individual, as variations in mandibular closing patterns or lack of stable occlusal dental stops may influence the number, location and intensity of dental contacts [42]. However, all participants included in this study presented a stable occlusion; thus, the potential variability related to occlusal instability was reduced. Other factors that may explain these findings include variations in the scanning procedure and differences in the occlusal-contact recording algorithms implemented in the software of the two evaluated systems.

The differences in agreement observed between the two digital occlusal recording workflows may reflect variations in the underlaying technologies, including the algorithms used to identify occlusal contacts, and the principles used to reconstruct interocclusal relationships and mandibular movements. The factors may influence the detection and assessment of occlusal contacts. However, the selection of an occlusal indicator and recording method should be guided by the clinical context and the clinician’s objectives. A comprehensive understanding of the technological and clinical factors that may influence occlusal registration is essential when interpreting digital occlusal findings.

A key methodological limitation of the present study was the use of 100 μm articulating paper as the physical reference comparator. The thickness and mechanical characteristics of an occlusal recording medium may affect mandibular closure behavior and, consequently, the number, size, and distribution of recorded markings. Thus, some of the observed differences between articulating paper and the digital workflows may reflect characteristics of the physical comparator rather than measurement differences associated with the digital workflows. In the present study, only a single articulating paper thickness was evaluated; therefore, the magnitude or direction of this potential effect could not be determined.

Another methodological consideration is that the two digital workflows were not completely independent. The maxillary and mandibular digital models acquired with the Medit i700 were imported into Tizian Function Pro 4.0.2 Software and aligned in MIP using the virtual interocclusal recording generated by the Medit Scan for Clinics v3.4.12 software. Therefore, the present study compared two digital clinical workflows rather than two fully independent acquisition methods. In addition, all participants underwent occlusal recordings in the same sequence, namely articulating paper, Medit i700 and Tizian JMA-Optic, with one hour interval between recordings. The sequence of occlusal recordings may represent another methodological consideration. All participants underwent the recordings in the same order, articulating paper, Medit i700 and Tizian JMA-Optic, with one hour interval between recordings. Consequently, patient related factors, including familiarization with the requested mandibular movements, fatigue, or short-term biological variation, cannot be completely excluded as potential contributors to differences between the occlusal recordings.

The absence of patient-reported occlusal perception represents an additional limitation of the present study. Subjective feedback regarding perceived occlusal contacts could have provided complementary information regarding the clinical relevance of the findings and the ability of the digital workflows to identify occlusal contacts that are clinically perceptible to patients. Furthermore, the low frequency of certain dynamic occlusal patterns limited the amount of independent information available for some categories. Although the number of tooth-level observations was relatively large, the effective sample size at the participant level was considerably smaller for sparse categories, resulting in limited precision of the corresponding estimates. Therefore, high point estimates in sparse categories, including values of 100%, should be interpreted cautiously. A particular limitation is that concordance between methods was assessed only at tooth level, based on the presence or absence of an occlusal contact. The precise intra-tooth correspondence of contact locations was not evaluated among the three recording methods. Thus, the level of concordance reported in this study should not be interpreted as indicating that the three methods identified contacts at identical locations on the occlusal surface.

Finally, this study included only young adults with complete permanent dentition, stable functional occlusion, and healthy temporomandibular joints. Therefore, the results should be interpreted within the characteristics of this population and should not be directly extrapolated to older patients, patients with severe tooth wear, extensive restorations or missing tooth, unstable occlusion, parafunction, temporomandibular disorders, parafunctional activity or complex prosthodontic rehabilitation.

The originality of this study lies in the clinical evaluation of two digital workflows, an intraoral scanner, Medit i700, and a jaw-tracking system, Tizian JMA-Optic, for static and dynamic occlusal assessment, including their reliability, diagnostic performance, and agreement with conventional articulating paper.

Further research should integrate spatial registration of contact locations to determine whether tooth-level agreement is also observed for the location, area, and distribution of occlusal contacts. Where appropriate, independent force- and timing-sensitive measurements could provide complementary data, allowing digital occlusal analysis workflows to be evaluated beyond the presence and absence of tooth-level contacts and providing additional insight into the characteristics of occlusal contact. The influence of occlusal indicator thickness should also be investigated by comparing multiple occlusal indicator thicknesses, including ultrathin foils, within the same participants. Because nominal and measured material thicknesses may differ, direct verification of the actual indicator thickness should also be considered in future methodological studies. Such an approach would help determine the extent to which physical indicator thickness contributes to concordance or discordance with digital occlusal-analysis workflows.

Nevertheless, future studies should investigate the potential effects of recording sequence by randomizing the order of recordings and using longer intervals between recordings, while also including more diverse patient populations. Studies should also evaluate different jaw-tracking systems integrated with intraoral scans acquired using various intraoral scanners in a more diverse populations to establish standardized protocols and to determine the accuracy, validity and clinical applicability of these novel digital technologies used for the assessment of the occlusal status. Such studies may help identify sources of variability among digital workflows and contribute to the development of standardized protocols for data acquisition and dental occlusal assessment.

## 5. Conclusions

The results indicated generally high intra-rater reliability for dynamic occlusal recordings, although reliability varied according to the occlusal-contact category and recording method. Both digital systems demonstrated good overall agreement with the articulating-paper reference comparator for quantifying occlusal contacts in MIP. Agreement varied according to dental area, being poor in the anterior areas and moderate in the posterior areas. Numerical differences between Medit i700 and Tizian JMA-Optic were not interpreted as evidence of superiority of either workflow. Although digital systems provide valuable quantitative information for occlusal analysis, articulating paper remains a widely used conventional method for clinical identification of occlusal contacts and served as the reference comparator in the present study. However, the findings should be interpreted in relation to the physical properties and 100 μm thickness of the indicator used. The present study was not designed to establish whether either digital workflow can replace articulating paper in routine clinical practice.

## Figures and Tables

**Table 1:** Intra-rater reliability of dynamic occlusal-contact recordings, with participant-cluster-bootstrap 95% confidence intervals.

Occlusion/Guidance	100 μm Articulating Paper, κ (95% CI)	Medit i700, κ (95% CI)	Tizian JMA-Optic, κ (95% CI)
P–CInc	1.000 (NE)	0.949 (0.820–1.000)	1.000 (NE)
P–CInc + LI	0.960 (0.909–1.000)	0.920 (0.806–0.985)	0.969 (0.922–1.000)
P–PPI	1.000 (NE)	0.908 (0.741–1.000)	1.000 (NE)
Right L–CG	0.973 (0.905–1.000)	1.000 (NE)	1.000 (NE)
Right L–AG	0.882 (0.539–1.000)	0.980 (0.937–1.000)	1.000 (NE)
Right L–GF	1.000 (NE)	0.886 (NE)	0.957 (0.883–1.000)
Right L–PLI	1.000 (NE)	0.883 (0.664–1.000)	0.688 (0.356–0.888)
Left L–CG	0.974 (0.914–1.000)	0.946 (0.857–1.000)	0.973 (0.910–1.000)
Left L–AG	0.795 (0.000–1.000)	0.704 (0.000–1.000)	0.869 (0.000–1.000)
Left L–GF	1.000 (NE)	1.000 (NE)	1.000 (NE)
Left L–PLI	1.000 (NE)	0.824 (0.651–0.949)	0.971 (0.903–1.000)

Cohen’s κ was calculated from tooth-level observations comparing the first and second recording sessions. Because multiple teeth were evaluated within each participant, 95% confidence intervals were obtained using participant-level cluster bootstrap resampling (10,000 replicates), with all tooth-level observations from each sampled participant retained together. NE, not estimable because of complete concordance or insufficient between-participant information in a sparse category. P, protrusion; CInc, central incisor; LI, lateral incisor; PPI, passive propulsive interference; L, laterotrusion; CG, canine guidance; AG, anterolateral guidance; GF, group function; PLI, passive laterotrusive interference.

**Table 2:** Tooth-level sensitivity and specificity of Medit i700 and Tizian JMA-Optic relative to the 100 μm articulating-paper reference method, with participant-cluster-adjusted 95% confidence intervals.

Occlusion/Guidance	Method	TP	FN	FP	TN	Sensitivity, % (95% CI)	Specificity, % (95% CI)
P–CInc	Medit i700	36	8	8	260	81.82 (43.20–98.50)	97.01 (89.30–99.70)
P–CInc	Tizian JMA-Optic	36	8	12	256	81.82 (43.20–98.50)	95.52 (87.40–99.10)
P–CInc + LI	Medit i700	70	13	20	209	84.34 (55.20–97.80)	91.27 (81.70–96.80)
P–CInc + LI	Tizian JMA-Optic	65	18	27	202	78.31 (48.60–95.30)	88.21 (77.20–95.10)
P–PPI	Medit i700	13	0	12	391	100.00 (NE)	97.02 (92.20–99.30)
P–PPI	Tizian JMA-Optic	9	4	20	383	69.23 (7.55–99.70)	95.04 (90.40–97.90)
Right L–CG	Medit i700	38	4	0	322	90.48 (68.10–99.00)	100.00 (NE)
Right L–CG	Tizian JMA-Optic	32	10	0	322	76.19 (51.40–92.40)	100.00 (NE)
Right L–AG	Medit i700	18	3	8	335	85.71 (48.40–99.20)	97.67 (91.50–99.80)
Right L–AG	Tizian JMA-Optic	21	0	12	331	100.00 (NE)	96.50 (90.00–99.30)
Right L–GF	Medit i700	8	2	0	354	80.00 (NE)	100.00 (NE)
Right L–GF	Tizian JMA-Optic	8	2	16	338	80.00 (NE)	95.48 (83.20–99.60)
Right L–PLI	Medit i700	8	0	10	346	100.00 (NE)	97.19 (91.60–99.50)
Right L–PLI	Tizian JMA-Optic	8	0	16	340	100.00 (NE)	95.51 (90.20–98.40)
Left L–CG	Medit i700	42	2	2	318	95.45 (75.70–99.90)	99.38 (96.40–100.00)
Left L–CG	Tizian JMA-Optic	40	4	2	318	90.91 (69.40–99.00)	99.38 (96.40–100.00)
Left L–AG	Medit i700	4	4	6	350	50.00 (0.00–100.00)	98.31 (93.40–99.80)
Left L–AG	Tizian JMA-Optic	4	4	10	346	50.00 (0.00–100.00)	97.19 (91.50–99.50)
Left L–GF	Medit i700	16	0	0	348	100.00 (NE)	100.00 (NE)
Left L–GF	Tizian JMA-Optic	16	0	4	344	100.00 (NE)	98.85 (93.20–100.00)
Left L–PLI	Medit i700	14	0	20	330	100.00 (NE)	94.29 (89.90–97.20)
Left L–PLI	Tizian JMA-Optic	8	6	31	319	57.14 (11.10–94.90)	91.14 (84.90–95.40)

Between-method analyses used the first recording session only. Tooth-level observations were clustered within participants for confidence-interval estimation. TP, true positive; FN, false negative; FP, false positive; TN, true negative; NE, not estimable because the relevant data provided insufficient between-participant information for estimation of a cluster-adjusted confidence interval. P, protrusion; CInc, central incisor; LI, lateral incisor; PPI, passive propulsive interference; L, laterotrusion; CG, canine guidance; AG, anterolateral guidance; GF, group function; PLI, passive laterotrusive interference.

**Table 3:** Positive and negative predictive values of Medit i700 and Tizian JMA-Optic relative to the 100 μm articulating-paper reference method, with participant-cluster-adjusted 95% CI.

Occlusion/Guidance	Method	PPV, % (95% CI)	NPV, % (95% CI)
P–CInc	Medit i700	81.82 (43.20–98.50)	97.01 (89.30–99.70)
P–CInc	Tizian JMA-Optic	75.00 (38.70–95.80)	96.97 (89.20–99.70)
P–CInc + LI	Medit i700	77.78 (55.50–92.30)	94.14 (82.20–99.00)
P–CInc + LI	Tizian JMA-Optic	70.65 (48.50–87.40)	91.82 (79.50–97.90)
P–PPI	Medit i700	52.00 (9.98–91.80)	100.00 (NE)
P–PPI	Tizian JMA-Optic	31.03 (8.02–64.30)	98.97 (96.10–99.90)
Right L–CG	Medit i700	100.00 (NE)	98.77 (95.50–99.90)
Right L–CG	Tizian JMA-Optic	100.00 (NE)	96.99 (93.40–99.00)
Right L–AG	Medit i700	69.23 (15.00–98.70)	99.11 (96.40–99.90)
Right L–AG	Tizian JMA-Optic	63.64 (16.40–96.10)	100.00 (NE)
Right L–GF	Medit i700	100.00 (NE)	99.44 (96.60–100.00)
Right L–GF	Tizian JMA-Optic	33.33 (0.00–100.00)	99.41 (96.40–100.00)
Right L–PLI	Medit i700	44.44 (6.46–88.60)	100.00 (NE)
Right L–PLI	Tizian JMA-Optic	33.33 (10.80–63.50)	100.00 (NE)
Left L–CG	Medit i700	95.45 (75.70–99.90)	99.38 (96.40–100.00)
Left L–CG	Tizian JMA-Optic	95.24 (74.60–99.90)	98.76 (95.50–99.90)
Left L–AG	Medit i700	40.00 (0.00–100.00)	98.87 (93.40–100.00)
Left L–AG	Tizian JMA-Optic	28.57 (0.00–98.90)	98.86 (93.40–100.00)
Left L–GF	Medit i700	100.00 (NE)	100.00 (NE)
Left L–GF	Tizian JMA-Optic	80.00 (0.00–100.00)	100.00 (NE)
Left L–PLI	Medit i700	41.18 (13.10–74.40)	100.00 (NE)
Left L–PLI	Tizian JMA-Optic	20.51 (2.91–55.10)	98.15 (94.60–99.60)

Between-method analyses used the first recording session only. Confidence intervals account for clustering of teeth within participants. NE, not estimable because the relevant data provided insufficient between-participant information for estimation of a cluster-adjusted confidence interval. PPV, positive predictive value; NPV, negative predictive value; P, protrusion; CInc, central incisor; LI, lateral incisor; PPI, passive propulsive interference; L, laterotrusion; CG, canine guidance; AG, anterolateral guidance; GF, group function; PLI, passive laterotrusive interference.

## Data Availability

Data Availability is available from the first author, upon request.
